# Using the Alcohol, Smoking and Substance Involvement Screening Test to predict substance‐related hospitalisation after release from prison: A cohort study

**DOI:** 10.1111/add.16365

**Published:** 2023-10-19

**Authors:** Craig Cumming, Stuart A. Kinner, Rebecca McKetin, Jesse T. Young, Ian Li, David B. Preen

**Affiliations:** ^1^ Centre for Health Services Research, School of Population and Global Health University of Western Australia Crawley Australia; ^2^ Centre for Adolescent Health Murdoch Children's Research Institute Parkville Australia; ^3^ Melbourne School of Population and Global Health The University of Melbourne Parkville Australia; ^4^ Griffith Criminology Institute Griffith University Mt Gravatt Australia; ^5^ School of Population Health Curtin University Perth Australia; ^6^ National Drug and Alcohol Research Centre University of New South Wales Sydney Australia; ^7^ Institute for Mental Health Policy Research Centre for Addiction and Mental Health Toronto Canada; ^8^ Centre for Epidemiology and Biostatistics, Melbourne School of Population and Global Health The University of Melbourne Parkville Australia; ^9^ National Drug Research Institute Curtin University Perth Australia; ^10^ School of Population and Global Health University of Western Australia Crawley Australia

**Keywords:** alcohol smoking and substance involvement test, hospitalisation, predictive validity, prison, risk, screening, substance use

## Abstract

**Background and Aims:**

Poor substance use‐related health outcomes after release from prison are common. Identifying people at greatest risk of substance use and related harms post‐release would help to target support at those most in need. The Alcohol Smoking and Substance Involvement Screening Test (ASSIST) is a validated substance use screener, but its utility in predicting substance‐related hospitalisation post‐release is unestablished. We measured whether screening for moderate/high‐risk substance use on the ASSIST was associated with increased risk of substance‐related hospitalisation.

**Design:**

A prospective cohort study.

**Setting:**

Prisons in Queensland and Western Australia.

**Participants:**

Participants were incarcerated and within 6 weeks of expected release when recruited. A total of 2585 participants were followed up for a median of 873 days.

**Measurements:**

Baseline survey data were combined with linked unit record administrative hospital data. We used the ASSIST to assess participants for moderate/high‐risk cannabis, methamphetamine and heroin use in the 3 months prior to incarceration. We used International Classification of Diseases (ICD) codes to identify substance‐related hospitalisations during follow‐up. We compared rates of substance‐related hospitalisation between those classified as low/no‐risk and moderate/high‐risk on the ASSIST for each substance. We estimated adjusted hazard ratios (aHR) by ASSIST risk group for each substance using Weibull regression survival analysis allowing for multiple failures.

**Findings:**

During follow‐up, 158 (6%) participants had cannabis‐related, 178 (7%) had opioid‐related and 266 (10%) had methamphetamine‐related hospitalisation. The hazard rates of substance‐related hospitalisation after prison were significantly higher among those who screened moderate/high‐risk compared with those screening low risk on the ASSIST for cannabis (aHR 2.38, 95% confidence interval [CI] 1.74, 3.24), methamphetamine (aHR 2.23, 95%CI 1.75, 2.84) and heroin (aHR 5.79, 95%CI 4.41, 7.60).

**Conclusions:**

Incarcerated people with an Alcohol Smoking and Substance Involvement Screening Test (ASSIST) screening of moderate/high‐risk substance use appear to have a significantly higher risk of post‐release substance‐related hospitalisation than those with low risk. Administering the ASSIST during incarceration may inform who has the greatest need for substance use treatment and harm reduction services in prison and after release from prison.

## INTRODUCTION

Illicit substance use is more prevalent among people entering prisons than in the general population [1, 2, 3, 4], particularly the use of cannabis, cocaine, amphetamines and heroin [3, 5]. Incarceration has a negligible impact on the risk of returning to substance use and overdose after release from prison [6, 7]. A range of preventable poor health outcomes, including fatal and non‐fatal overdose, are common in the period soon after release from prison [6, 8] and can lead to increased contact with emergency departments (EDs) and hospitals [9, 10]. This increased ED contact and hospitalisation after release from prison comes at a substantial economic cost [11, 12]. Given this, one priority for healthcare practitioners in prison is being able to identify those at greatest risk of harmful substance use so that interventions aimed at preventing or reducing substance use and its related harms can be effectively targeted.

One screening tool that can be used to identify individuals at risk of poor substance use outcomes after release from prison is the World Health Organization Alcohol, Smoking and Substance Involvement Screening Test (ASSIST) [13]. The ASSIST is an eight item screening tool used to assess patients for moderate or high‐risk substance use that may require treatment. It has good concurrent, construct and discriminant validity in community substance treatment and healthcare settings [14, 15]. In prison settings, the ASSIST has demonstrated good test–retest reliability and good concurrent validity in comparison with the items measuring substance use disorder in the Structured Clinical Interview for Diagnostic and Statistical Manual of Mental Disorders (DSM)‐IV‐Non‐Patient Version with Psychotic Screen (SCID) [16, 17].

The ASSIST has also been found to have good accuracy at predicting substance use in the 6 months after release from prison [18]. However, to date there is no research investigating how well the ASSIST predicts hospitalisation related to substance use after release from prison. Therefore, we aimed to investigate the association between ASSIST scores that assessed substance use patterns in the 3 months before incarceration and substance‐related hospitalisation after release from prison. We specifically investigated cannabis, methamphetamine and opioid/heroin‐related hospitalisation, because the use of these substances is highly prevalent among people entering prison in Australia [3].

## METHODS

This cohort study combined in‐prison survey data with prospectively linked administrative ED and inpatient hospital records after release from prison. Our primary exposure was pre‐incarceration substance use, and our primary outcome was substance‐related hospitalisation.

### Participants and procedure

**FIGURE 1:**
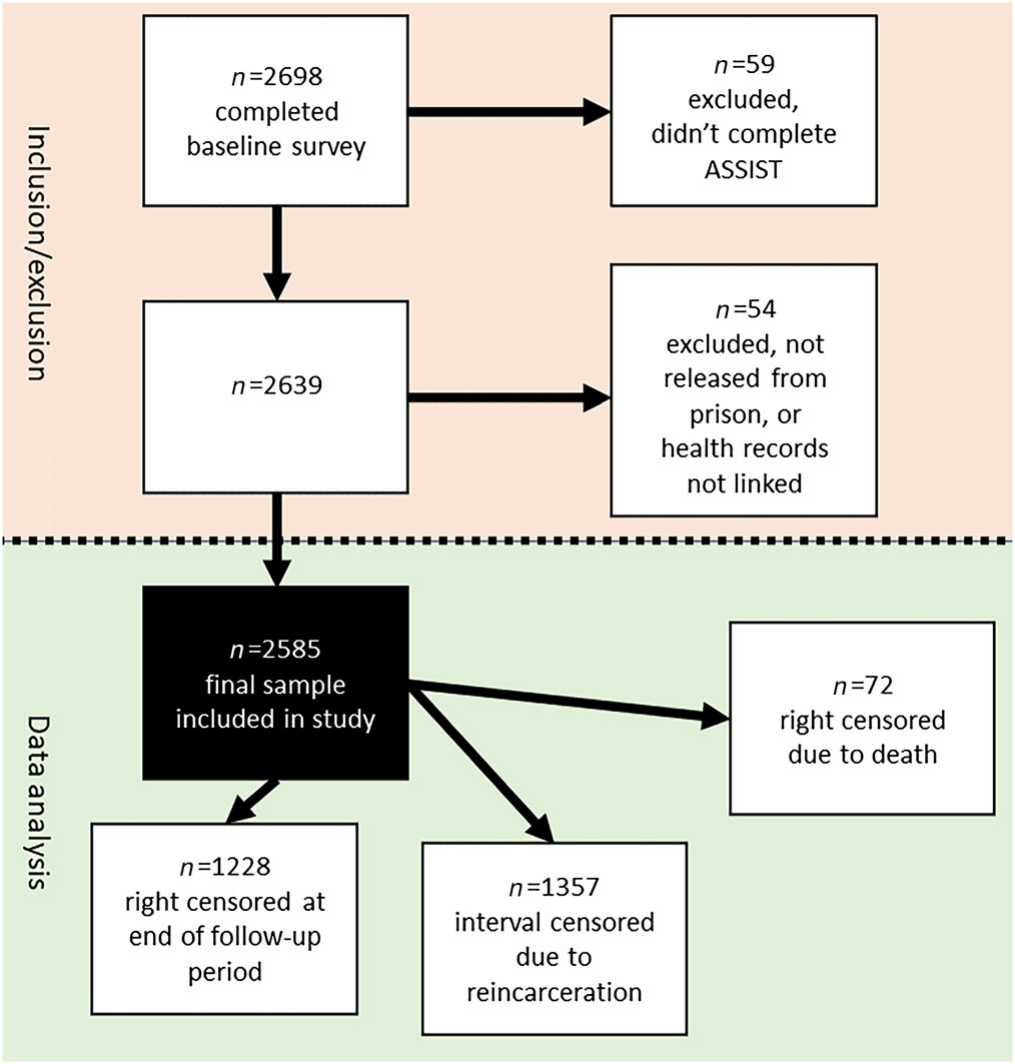
Flow chart for participant inclusion and exclusion.

Participants were adults (≥18 years) recruited within 6 weeks of their expected release from prison in two Australian states: Queensland (QLD) (between 2008 and 2010) and Western Australia (WA) (between 2013 and 2016). On recruitment, participants completed a baseline health survey that took 60 to 90 minutes to complete and consented to their administrative ED and inpatient hospital records and their correctional data being linked for a period of up to 5 years either side of their index incarceration (the period of incarceration when they were recruited). Our study focused on the period after each participant's release from their index incarceration (the period of incarceration during which they were recruited). Follow‐up for each participant started on the date of release from their index incarceration and ended either at the earlier of date of death, or 31 July 2012 (QLD participants), or 30 June 2018 (WA participants). Cohort members included in this study had to have: (1) completed the baseline (in prison) survey including the ASSIST items for at least one of the three substances being investigated (cannabis, methamphetamine, heroin); (2) their administrative health data linked; and (3) been released from incarceration at least once during follow‐up (see Figure 1).

### Data sources

#### Baseline survey and covariates

Sociodemographic and health variables included age, sex (inferred from the prison where recruitment occurred), indigeneity, length of index incarceration, whether participants were removed from their family as children, employment status in the 6 months before index incarceration, a prior mental illness diagnosis, history of injecting drug use and history of overdose. All sociodemographic variables were taken from the baseline survey except for index incarceration length, which was taken from linked administrative corrections records. Most variables were dichotomous (yes/no) with index incarceration length trichotomous (≤90 days, 91–365 days and >365 days) [19] and age was a discrete numerical variable.

##### Primary exposure: ASSIST

Our primary exposure of interest was pre‐incarceration substance use. At baseline, we used the ASSIST to assess participants' use of cannabis, methamphetamine and heroin in the 3 months before reception into prison. For each substance, the raw ASSIST score (possible range = 0–39) was classified as either low/no‐risk (<4), moderate risk (≥4 and <27), or high‐risk (≥27) [13]. During the development of the ASSIST, the moderate and high risk ASSIST groups were designed to align with the diagnoses ‘substance abuse’ and ‘substance dependence’, respectively in DSM‐IV [15], both of which are now encompassed by ‘substance use disorder’ in the newer DSM‐5 [20]. Accordingly, we dichotomised participants into low/no risk and moderate/high risk groups to align with the single DSM‐5 substance use disorder category as has been done previously [19, 21].

##### K10

The baseline survey also included the 10‐item Kessler psychological distress scale (K10) (score range = 10–50) [22], which was used to assess participants for psychological distress at baseline. Participants were dichotomised into low/no distress (≤15) and moderate/high distress [16, 17, 18, 19, 20, 21, 22, 23, 24, 25, 26, 27, 28, 29, 30, 31, 32, 33, 34, 35, 36, 37, 38, 39, 40, 41, 42, 43, 44, 45, 46, 47, 48, 49, 50] consistent with the Australian National Health Survey [23].

##### ESSI

The Enrichd Social Support Inventory (ESSI) [24] is a seven item screening tool (scoring range = 8–34), which was used to assess participants for level of social support available at baseline, with participants dichotomised at the median (<28/≥28) as has been done previously [18].

#### Administrative linked data

##### Outcomes: Hospital records

Our primary outcome of interest was substance‐related ED presentations and inpatient hospital admissions (together referred to as ‘hospitalisation’). Unit record ED presentations and inpatient hospital admissions for each participant were obtained from state‐wide collections in QLD and WA. Inpatient hospital admission records included date of admission, primary diagnosis, up to 48 secondary diagnosis codes and up to four external cause codes, using the International Classification of Disease, 10th revision, Australian Modification (ICD‐10‐AM) [25]. ED records included date of presentation and one ICD‐10‐AM code representing the principal discharge diagnosis. For this outcome variable, we identified ‘specific‐substance‐related’ records (hospitalisation relating to each substance specifically) from ICD‐10‐AM codes: cannabis‐related (F12), methamphetamine‐related (F15, T43.6), opioid‐related (F11, T40.1–T40.4). We also identified a broader category of ‘any‐substance‐related’ records (related to the use of any substance, not just cannabis, methamphetamine or opioids) (F10–F19, X40–X45, X60–X64, X85, T36–T51, Y10–Y15) [19]. We considered ICD‐10‐AM codes relating to psychostimulant use a proxy for methamphetamine‐related hospital use, as this has previously been found to have acceptable accuracy [26]. For heroin‐related hospital use, we relied on ICD‐10‐AM codes relating to opioid use as 84% of participants who screened for moderate/high risk heroin use on the ASSIST reported ever using other opioids, there are few heroin‐specific ICD‐10‐AM codes, and the evidence suggests that heroin users are highly likely to also use other opioids [27, 28, 29].

##### Corrections and death records

Corrections records were obtained from the QLD Department of Corrective Services and WA Department of Justice for index incarceration and subsequent reincarceration during follow‐up. We censored for deaths using National Death Index records, which were obtained from the Australian Institute of Health and Welfare and included date of death data for all deaths occurring during follow‐up.

### Statistical analysis

Four separate time‐periods were modelled, all starting from the time of discharge from index incarceration (earliest index discharge dates 11 September 2008 for QLD participants, 30 May 2013 for WA participants), and continuing until either 3, 6 or 12 months later, or the end of the study period (31 July 2012 for QLD participants, 30 June 2018 for WA participants). Interval‐censoring was used to account for periods of reincarceration. Follow‐up time for participants who died before the end of the relevant follow‐up period was right‐censored at date of death. Substance‐related hospitalisation that occurred during periods of incarceration (*n* = 35) were excluded from the analysis. Multiple substance‐related hospitalisations that occurred for the same participant on the same day were assumed to relate to the same substance‐related event and were combined as a single hospitalisation, resulting in 2491 substance‐related hospital events being consolidated to 2226 events. We also calculated the rate of specific‐substance‐related and any‐substance‐related hospitalisation across all participants during the four time periods. We performed *post hoc* analysis and calculated the rates of days reincarcerated, and the mortality rates for the moderate/high risk participants for each substance to assess how this may have impacted time‐at‐risk and changes in hospitalisation rates during follow‐up.

Sociodemographic and health‐related characteristics potentially associated with post‐incarceration substance use resulting in hospitalisation were identified from the literature [7, 9, 30, 31, 32, 33, 34, 35, 36, 37] and were taken from the baseline survey as described earlier. For each characteristic, we compared the relative risk between groups (with and without the characteristic) of having a hospitalisation related to the use of any drug during the entire follow‐up period using modified log‐linked Poisson regression with robust error variance [38]. The single exception to this was age, which was modelled as a continuous variable using linear regression.

To address our main aim, we conducted both unadjusted and adjusted survival analysis using Weibull regression [39], allowing for multiple failures per participant, to compare the hazard rates of specific‐substance‐related and any‐substance‐related hospitalisation for participants in the low/no and moderate/high ASSIST risk groups (for cannabis, methamphetamine and heroin) at each time point during follow‐up. In the multivariable models, we adjusted for all sociodemographic and health covariates described above. The rationale for including these covariates and how we handled them is described in more detail in the Supporting Information.

To investigate the possibility of selection bias resulting from inclusion and exclusion criteria, we investigated whether (1) a participant being classified as moderate/high risk using the ASSIST across any substance (cannabis, methamphetamine or heroin); or (2) the sociodemographic/health covariates predicted being excluded from the study. We conducted sensitivity analysis to investigate the possibility of bias resulting from our assumption that multiple substance‐related hospitalisations occurring on the same day for a participant resulted from the same substance‐related event. Our methods and results of these additional analyses are described in detail in the Supporting Information (p. 1). There were some missing covariate data, which resulted in a small number of participants (described below) being dropped from the multivariable analyses. We attempted to use multiple imputation to impute these missing values and re‐run the analyses using the imputed data, however, the missing covariate data were ‘missing not at random’, which risks generating misleading results if multiple imputation is used [40], therefore, we elected not to do this in light of the small amount of missing data.

All statistical analyses were performed using Stata version 17 [41].

## RESULTS

### Participants and follow‐up

Of participants who completed the baseline survey (*n* = 2698), 2585 (96%) were included in the study after the exclusion criteria were applied. A total of 2573 (99.5%) completed the ASSIST for all three substances (2583 for cannabis, 2581 for methamphetamine and 2573 for heroin). Included participants were followed up in the community for 6057 person‐years, for a median of 873 (IQR= 635, 1117; range = 6–1851) days per person. A total of 52% (*n* = 1357) of participants had their follow‐up time interval‐censored because of reincarceration, resulting in 1513 person‐years being interval‐censored, and 72 (3%) participants who were censored because of death. Death rates during the entire follow‐up period for the moderate/high risk groups for each substance were similar for cannabis (12.1 per 1000 person‐years, 95% CI = 9.0, 16.0) and methamphetamine (12.6 per 1000 person‐years, 95% CI = 9.3, 16.8) with heroin higher at 22.4 per 1000 person‐years (95% CI = 14.6, 32.8). Median days reincarcerated during follow‐up for the moderate/high risk groups were as follows: cannabis, 105 days; methamphetamine, 140 days; and heroin, 189 days. Missing covariate data resulted in 30 (1%) participants being dropped from the multivariable cannabis (*n* = 2553) and methamphetamine (*n* = 2551) analyses, and 28 (1%) participants being dropped from the multivariable opioid/heroin (*n* = 2545) analyses.

### Baseline sociodemographic characteristics and ASSIST scores

There were 517 (20%) females, 936 (36%) Indigenous participants and the median age was 29 years (IQR = 23, 36) at baseline (Table 1). The majority (*n* = 1335) had an index incarceration of between 91 and 365 days with 772 (30%) incarcerated for more than a year. A total of 53% (*n* = 1372) of participants screened positive for moderate/high risk cannabis use, 1279 (50%) for moderate/high risk methamphetamine use and 389 (15%) for moderate/high risk heroin use.

**TABLE 1:** Potential baseline predictors of any‐substance‐related hospital or emergency department use during follow‐up.

Baseline characteristics	Any drug‐related hospital or ED use during follow‐up		*P*	Total participants (%)
	*n* (%)	RR (95% CI)		
Cannabis (ASSIST)				
Low/no risk cannabis use (ref)	273 (23)	–	–	1211 (47)
Moderate/high risk cannabis use	476 (35)	1.53 (1.35, 1.74)	<0.001	1372 (53)
Methamphetamine (ASSIST)				
Low/no risk methamphetamine use (ref)	309 (24)	–	–	1302 (50)
Moderate/high risk methamphetamine use	439 (34)	1.45 (1.28, 1.64)	<0.001	1279 (50)
Heroin (ASSIST)				
Low risk heroin use (ref)	569 (26)	–	–	2184 (85)
Moderate/high risk heroin use	177 (46)	1.75 (1.54, 2.00)	<0.001	389 (15)
Sex				
Male (ref)	544 (26)	–	–	2068 (80)
Female	206 (40)	1.51 (1.33, 1.72)	<0.001	517 (20)
Indigeneity				
Indigenous	373 (23)	1.78 (1.58, 2.00)	<0.001	936 (36)
Non‐Indigenous	377 (50)			1649 (64)
Age at baseline (vs 1 year younger)^a^ linear regression coefficient	–	−0.0002 (−0.0018, 0.0014)^a^	0.868	Median = 27 IQR = 23, 34
Index incarceration length				
≤90 days	138 (29)	1.04 (0.87, 1.25)	0.660	478 (18)
91–365 days	404 (30)	1.11 (0.96, 1.27)	0.160	1335 (52)
>365 days (reference)	208 (27)	–	–	772 (30)
Employed pre‐index incarceration				
Unemployed (ref)	536 (35)			1514 (59)
Employed	214 (20)	0.56 (0.49, 0.65)	<0.001	1071 (41)
Ever taken from family as a child				
Not taken (ref)	587 (28)	–	–	2117 (82)
Taken	161 (35)	1.27 (1.10, 1.46)	0.001	459 (18)
Psychological distress (K10)				
Low/no distress (ref)	318 (43)			1249 (49)
Moderate/high distress	429 (32)	1.27 (1.13, 1.44)	<0.001	1323 (51))
Mental illness diagnosed (lifetime)				
Never diagnosed (ref)	344 (24)	–	–	1422 (55)
Ever diagnosed	406 (35)	1.44 (1.28, 1.63)	<0.001	1162 (45)
Injecting drug use (lifetime)				
Never injected drugs (ref)	202 (19)	–	–	1090 (42)
Ever injected drugs	546 (37)	1.97 (1.71, 2.27)	<0.001	1493 (58)
Drug overdose (lifetime)				
Never overdosed (ref)	507 (26)			1954 (76)
Ever overdosed	240 (39)	1.50 (1.32, 1.69)	<0.001	618 (24)
Social support (ESSI)				
Moderate/high social support (ESSI = 28+) (ref)	370 (27)	–	–	1385 (54)
Low social support (ESSI <28)	380 (32)	1.19 (1.05, 1.34)	0.006	1200 (46)

Abbreviations: ASSIST, Alcohol Smoking and Substance Involvement Screening Test; ESSI, Enrichd Social Support; K10, Kessler Psychological Distress Scale; ref, reference group, RR, relative risk.

^a^ Linear regression coefficient.

### Hospitalisation post‐release

A total of 29% (*n* = 750) of participants had at least one hospitalisation related to substance use during follow‐up. A total of 35% (*n* = 476) of the moderate/high risk cannabis group, 34% of the moderate/high risk methamphetamine (*n* = 439) group and 46% (*n* = 177) of the moderate/high risk heroin group had at least one any‐substance‐related hospitalisation during follow‐up. A total of 6% (*n* = 158) of participants had at least one cannabis‐related hospitalisation, 266 (10%) had at least one methamphetamine‐related hospitalisation and 178 (7%) had at least one opioid‐related hospitalisation during follow‐up.

#### Hospitalisation rates

**FIGURE 2:**
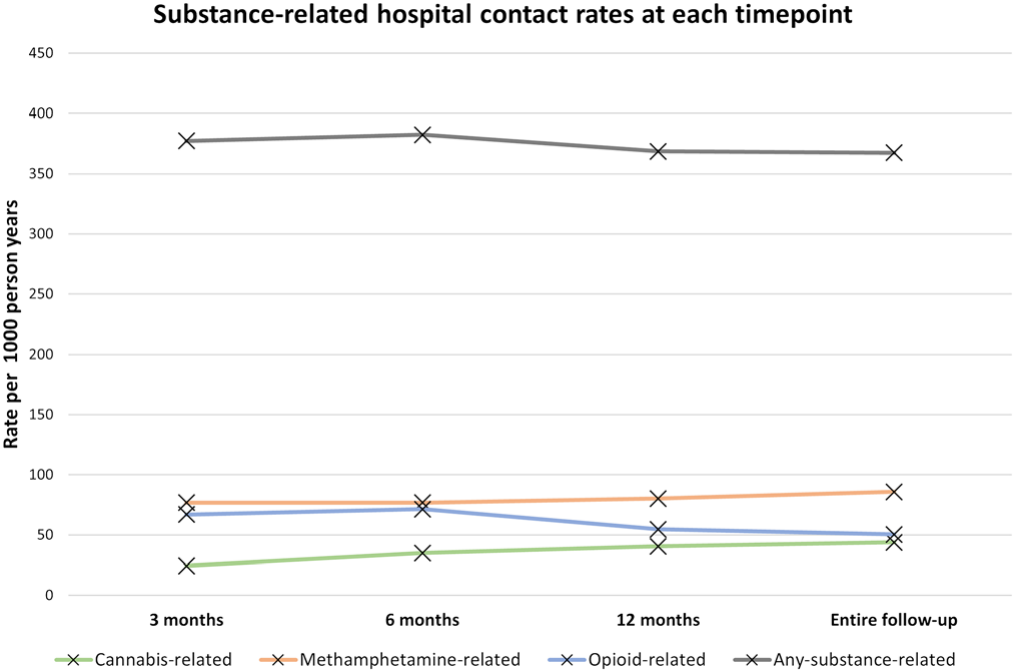
Substance‐related hospital contact rates per 1000 person‐years, stratified by substance type.

During follow‐up there were 2226 hospitalisations related to any substance use at a rate of 367.5 per 1000 person‐years (Table 2). For specific‐substance‐related hospitalisation, methamphetamine was the most frequent with 520 events at a rate of 85.9 per 1000 person‐years, followed by opioids with 305 events at 50.4 per 1000 person‐years, then cannabis with 265 events at 43.8 per 1000 person‐years. With respect to changes in rates over time, cannabis‐related hospitalisation increased from 24.5 per 1000 person‐years at 3 months post‐release to 43.8 per 1000 person‐years across the entire follow‐up period (Figure 2). Conversely, opioid‐related hospitalisation decreased from 67.0 to 50.4 per 1000 person‐years across the same time periods, with methamphetamine increasing slightly from 76.8 per 1000 person‐years at 3 months to 85.9 during the entire follow‐up period.

**TABLE 2:** Number of drug‐related hospital or ED events and event rates per 1000 person years stratified by ASSIST risk group for each drug.

Time period	Cannabis		Methamphetamine		Opioid		Any drug	
	Count	Rate (95% CI)	Count	Rate (95% CI)	Count	Rate (95% CI)	Count	Rate (95% CI)
3 months								
Low/no risk	2	7.0 (1.7, 27.8)	6	19.2 (8.6, 42.8)	7	13.5 (6.4, 28.3)		
Mod/hi risk	13	40.1 (23.3, 69.1)	41	137.1 (101.0, 186.2)	34	377.1 (269.4, 527.7)		
Total	15	24.5 (14.8, 40.7)	47	76.8 (57.7, 102.2)	41	67.0 (49.3, 91.0)	231	377.4 (331.8, 429.4)
6 months								
Low/no risk	7	12.6 (6.0, 26.4)	13	21.5 (12.5, 37.2)	17	17.0 (10.6, 27.4)		
Mod/hi risk	34	55.2 (39.4, 77.3)	77	135.7 (108.6, 169.7)	55	329.0 (252.6.7, 428.5)		
Total	41	35.0 (25.8, 47.5)	90	76.8 (62.5, 94.4)	72	61.4 (48.8, 77.4)	448	382.4 (348.5, 419.4)
12 months								
Low/no risk	16	15.0 (8.6, 24.3)	30	26.0 (18.2, 37.2)	35	18.4 (13.2, 25.7)		
Mod/hi risk	74	65.9 (51.9, 82.5)	148	139.9 (119.1, 164.4)	86	308.2 (225.9, 344.7)		
Total	90	40.6 (33.0, 49.9)	178	80.3 (69.3, 93.0)	121	54.6 (45.7, 65.2)	817	368.6 (344.2, 394.8)
Entire follow‐up period								
Low/no risk	56	19.1 (14.7, 24.8)	107	33.0 (27.1, 39.6)	101	21.8 (19.5, 23.7)		
Mod/hi risk	209	67.0 (58.5, 76.8)	411	147.6 (134.0, 162.6)	204	242.1 (211.1, 277.7)		
Total	265	43.8 (38.8, 49.4)	520	85.9 (78.8, 93.6)	305	50.4 (45.0, 56.3)	2226	367.5 (352.6, 383.1)

#### Comparing risk of hospitalisation between low/no and moderate/high ASSIST risk groups

In our unadjusted analyses, the hazard rates for participants in each of the moderate/high risk groups was multiple times higher for specific‐substance related hospitalisation compared to their low/no risk counterparts, with the biggest difference across the follow‐up period observed for heroin (hazard ratio [HR] = 12.43; 95% CI = 9.79, 15.77) and the smallest observed for cannabis (HR = 3.52; 95% CI = 2.62, 4.73) (Table 3). The greatest significant difference in our adjusted hazard rates between the low/no risk and moderate/high risk groups was observed at 6 months for cannabis (adjusted hazard ratio [aHR] = 2.99; 95% CI = 1.30, 6.88) and methamphetamine (aHR = 3.08; 95% CI = 1.61, 5.87), and 3 months for heroin (aHR = 19.95; 95% CI = 8.20, 48.54). After adjusting for covariates, our estimates attenuated substantially toward the null, with the CIs tightening considerably, indicating underlying confounding in the unadjusted estimates. Our full multivariable models are provided in the Supporting Information (Tables S3–S26).

**TABLE 3:** Poisson regression comparing rates of specific‐substance‐related and any‐substance‐related hospital contact after release from prison for low/no and moderate/high risk ASSIST groups for each substance.

	Cannabis‐related		Methamphetamine‐related		Opioid‐related		Any substance‐related	
	HR (95% CI)	aHR^a^ (95% CI)	HR (95% CI)	aHR^a^ (95% CI)	HR (95% CI)	aHR^a^ (95% CI)	HR (95% CI)	aHR^a^ (95% CI)
3 months								
Cannabis	5.78 (1.30, 25.60)	3.72 (0.83, 16.70)					1.42 (1.09, 1.85)	0.97 (0.74, 1.28)
Methamphetamine			7.12 (3.02, 16.78)	3.14 (1.24, 7.95)			1.14 (0.88, 1.48)	0.60 (0.45, 0.81)
Heroin					27.84 (12.34, 62.81)	19.95 (8.20, 48.54)^b^	2.25 (1.69, 2.99)	1.53 (1.11, 2.11)
6 months								
Cannabis	4.40 (1.95, 9.93)	2.99 (1.30, 6.88)					1.74 (1.43, 2.11)	1.23 (1.01, 1.51)
Methamphetamine			6.29 (3.49, 11.32)	3.08 (1.61, 5.87)			1.38 (1.15, 1.67)	0.75 (0.60, 0.93)
Opioid					19.20 (11.15, 33.08)	8.15 (4.50, 14.75)	2.05 (1.66, 2.54)	1.32 (1.04, 1.67)
12 months								
Cannabis	4.32 (2.52, 7.41)	2.98 (1.70, 5.20)					1.89 (1.64, 2.19)	1.39 (1.19, 1.62)
Methamphetamine			5.39 (3.64, 7.98)	3.00 (1.93, 4.66)			1.38 (1.20, 1.59)	0.82 (0.70, 0.96)
Heroin					15.01 (10.13, 22.23)	6.33 (4.09, 9.79)	1.79 (1.52, 2.11)	1.28 (1.07, 1.54)
Entire follow								
Cannabis	3.52 (2.62, 4.73)	2.38 (1.74, 3.24)					1.89 (1.73, 2.06)	1.35 (1.23, 1.48)
Methamphetamine			4.51 (3.65, 5.58)	2.23 (1.75, 2.84)			1.41 (1.30, 1.53)	0.82 (0.74, 0.90)
Heroin					12.43 (9.79, 15.77)	5.79 (4.41, 7.60)	1.60 (1.44, 1.77)	1.12 (0.99, 1.25)

Abbreviations: aHR, adjusted hazard ratio; ASSIST, Alcohol Smoking and Substance Involvement Screening Test; HR, hazard ratio.

^a^ Adjusted for sex, indigeneity, age, employment status, being taken into care as a child, distress, mental illness diagnosis, injecting drug use, overdose, and low social support.

^b^ Lifetime injecting drug use variable removed from the model because of collinearity.

In our analysis of possible selection bias resulting from our inclusion and exclusion criteria, females were significantly less likely to be excluded than males (relative risk [RR] = 0.53; 95% CI = 0.30, 0.94) and Indigenous participants were more likely to be excluded than non‐Indigenous participants (RR = 2.22; 1.54, 3.19) (Table S1). There was no difference in risk of being excluded from the study for individuals screening for moderate/high risk of any of the three substances compared to those who screened low/no risk across all three substances (RR = 1.01; 95% CI = 0.61, 1.68). Our sensitivity analysis that did not combine multiple substance‐related hospitalisations occurring on the same day for the same participant into a single hospitalisation supported our main analysis (Table S2).

## DISCUSSION

Our study is the first to investigate whether the ASSIST may be a useful predictor of substance use resulting in hospitalisation following prison release. We found that participants screening for moderate/high risk cannabis, methamphetamine or heroin use were at significantly higher risk of having at least one episode of specific‐substance‐related and any‐substance‐related hospitalisation at 3, 6 and 12 months post‐release and during the entire follow‐up period compared to those who screened low/no risk. Our results suggest that the ASSIST, assessing pre‐incarceration patterns of substance use, administered during incarceration, can be used to identify a substantial number of individuals who may be at risk of poor substance‐related health outcomes after prison release, and may be a useful predictor of any‐substance‐related hospitalisation, and a strong predictor of specific‐substance‐related hospitalisation.

Our results provide further evidence of the substantial risk of returning to harmful substance use among people released from prison and are consistent with emerging evidence that incarceration is better conceived of as an interruption to substance use, rather than serving to prevent future use [6, 7, 18, 32]. More alcohol and other drug (AOD) treatment and support (including harm reduction interventions) commencing during incarceration, and continuing post release, is urgently needed to address this. Further, our findings that sociodemographic characteristics such as being female [6, 42], pre‐incarceration unemployment [32, 43], having a history of mental illness, injecting drug use and overdose [9], were associated with substance‐related hospitalisation is consistent with previous evidence. These factors should be considered for inclusion in risk assessment tools used by correctional health services to identify individuals who should be prioritised for AOD throughcare treatment and support.

Predicting harmful substance use is a key public policy priority, with substantial resources invested in investigating how neurobiology and technology could be used to predict future substance use [44], and what role factors such as mental illness [45], impulsivity, [46, 47] and traumatic childhood experiences [48] may play in this. This research is crucial in advancing knowledge in this area; however, prison settings often have resource constraints, and simple, cost‐effective options for assessing risk of future substance use (particularly high‐risk substance resulting in hospitalisation) that can be implemented at scale are needed. Given that the ASSIST is simple, quick and cost‐effective to administer in prison settings [17], our findings suggest that the ASSIST is a useful tool for assessing people for risk of substance use resulting in hospitalisation after release from prison. Being able to ascertain who is at an increased risk of substance‐related hospitalisation after release from prison, before prison release, is crucial in ensuring that both prison‐based and community‐based services are effectively targeted at those who need them most to prevent poor substance‐related health outcomes.

Our study is the first to examine the predictive validity of the ASSIST with respect to substance use resulting in hospitalisation after release from prison by using records of ‘real world’ acute health service contact obtained from state‐wide ED and hospital records. Our study builds on previous work that found that the ASSIST, administered in a prison setting before release, was a good predictor of a return to substance use during the first 6 months after release [18]. Our current study expands on these findings, establishing that the ASSIST can be used to predict who is at an increased risk of engaging in high‐risk substance use requiring treatment in hospital after release from prison. Previous research [14, 15, 49] had been limited to psychometric and clinical diagnostic measures to investigate the validity of the ASSIST, primarily comparing the performance of the ASSIST to other measures such as the Mini‐International Neuropsychiatric Interview [50], Addiction Severity Index [51], Severity of Dependence Scale [52], the Drug Abuse Screening Test [53] and the Rating of Injection Site Condition Scale [54]. Newcombe *et al*. [15] also investigated the ASSIST as a predictor of future substance use; however, they were limited by their sample size of 20 for this part of their study. In contrast to previous research, our findings provide evidence of hospital use resulting from substance use rather than behaviours or other factors that may indicate a substance use disorder. Importantly, our findings show that the ASSIST can be used to predict which people leaving prison are at high risk of substance use resulting in health outcomes severe enough to warrant treatment in acute healthcare settings. Therefore, this information can be used for targeting limited AOD treatment resources at people with the highest risk of poor health AOD‐related health outcomes, to reduce the number of people leaving prison that end up in the ED and hospital beds because of high‐risk substance use.

Our finding that opioid‐related hospitalisation rates dropped substantially between 3 and 12 months, as well as across the entire follow‐up period is not surprising. This pattern may be due in part to a reduction in opioid use [55] and/or tolerance for opioids occurring during incarceration [56, 57], resulting in an increased risk of overdose soon after release from prison. This risk may possibly subside over time as individuals access effective treatments such as medication‐assisted treatment (MAT) in the community. It could also possibly be because of the higher mortality rate observed for this group during follow‐up, although this result should be interpreted with caution, because our groups were not mutually exclusive. Long‐acting injectable buprenorphine is the current gold standard for treating opioid dependence and is strongly recommended for use in custodial settings [58, 59] to help prevent poor opioid‐related health outcomes during incarceration and after release. The ASSIST may be a useful tool for informing service providers who is most in need of this treatment.

To date, opioids have been a major focus of research, policy and treatment for those in prison [58, 59, 60]. However, the high rates of methamphetamine‐related hospitalisation we observed throughout follow‐up echo evidence elsewhere that methamphetamine use is now also an important driver of poor outcomes such as hospitalisation [61, 62] and deaths [63] in different parts of the world. The consistently high rates of methamphetamine‐related hospitalisation we observed across follow‐up may also be reflective of the fact that there is currently no effective pharmacotherapy to manage methamphetamine withdrawal [64], or to treat methamphetamine use disorder and that modest efficacy has been observed for psychosocial therapy [65]. Accordingly, there is an urgent need to develop effective pharmacotherapy treatment for treating methamphetamine use disorders, with work in this space ongoing [66]. Once developed, the ASSIST could be a useful tool for effectively targeting pharmacotherapy treatment at those at highest risk of methamphetamine‐related health problems after prison.

Our findings provide two clear messages for policymakers and practitioners at the intersection of the justice and health systems. First, the now overwhelming evidence of the heightened risk that people leaving prison face of returning to substance use (and the poor outcomes associated with this) is not just present for the initial period after release from prison, but likely persists for a longer period of potentially up to several years. Given the multitude of complex health and social challenges that this group faces [21, 67, 68], multidisciplinary health treatment and support commencing during incarceration and continuing post‐release is essential to reduce the risks of a return to substance use and poor associated outcomes. This is equally important for both the justice system, which often sees individuals who return to substance use cycle back through the system [69], and the health system, because it is already‐strained public hospitals and EDs that are impacted by substance use requiring medical treatment. Second, the ASSIST is a reliable and useful tool for those working in the justice system that can help identify people in their care who may be at high risk of returning to substance use after release prison. This is crucial as given the limited health resources available in prison settings [70, 71], it is vital that these are targeted at those most in need. With the ASSIST proven to be both suitable and cost‐effective to administer in prison settings [17, 72], there is a strong case for its use to become standard in all prisons, to help identify individuals early in their period of incarceration who warrant AOD treatment and support.

Our cohort study design and use of both baseline survey and linked administrative hospital and ED follow‐up data was a key strength of our study. This limited the loss to follow‐up that is a common challenge for studies involving people released from prison. This enabled us to compare the health outcomes after release from prison for participants with different patterns of pre‐incarceration substance use. Although one of the largest of its kind, the size of our sample was still a limitation and meant that some of our estimates lacked precision, particularly at our shorter follow‐up time points; replicating our study with a larger sample would assist in producing more precise estimates. Our analysis investigating potential selection bias showed that males and Indigenous people were more likely to be excluded from the study than their counterparts. This did not meaningfully impact our findings as the Indigenous proportion of participants in our study sample is still broadly consistent with that in the total population in QLD and WA [73], and females were intentionally oversampled to preserve statistical power. Although our sample only included participants from two Australian jurisdictions (so generalisation of our findings across Australia may be limited), these jurisdictions manage 39% of Australia's prisoner (and 47% of the Indigenous prisoner) population [74]. Our analysis plan was not pre‐registered so our findings should be considered exploratory.

## CONCLUSION

The ASSIST is a useful predictor of substance‐related hospitalisation after release from prison and can be easily and effectively used in prison settings. More AOD and health treatment and support is urgently needed to reduce the risk of a return to harmful substance use after release from prison.

## AUTHOR CONTRIBUTIONS

**Craig Cumming:** Conceptualization (lead); data curation (supporting); formal analysis (lead); investigation (lead); methodology (lead); project administration (equal); software (lead); writing—original draft (lead); writing—review and editing (lead). **Stuart A. Kinner:** Conceptualization (supporting); data curation (equal); formal analysis (supporting); funding acquisition (lead); investigation (supporting); methodology (supporting); project administration (equal); resources (equal); supervision (equal); writing—review and editing (supporting). **Rebecca McKetin:** Conceptualization (supporting); investigation (supporting); methodology (supporting); supervision (equal); writing—review and editing (supporting). **Jesse T. Young:** Conceptualization (supporting); data curation (supporting); formal analysis (supporting); investigation (supporting); methodology (supporting); project administration (supporting); software (supporting); writing—review and editing (supporting). **Ian Li:** Investigation (supporting); methodology (supporting); supervision (equal); writing—review and editing (supporting). **David B. Preen:** Conceptualization (supporting); data curation (supporting); funding acquisition (lead); investigation (supporting); methodology (supporting); project administration (supporting); resources (lead); supervision (lead); writing—original draft (supporting); writing—review and editing (supporting).

## DECLARATION OF INTERESTS

There are no competing interests.

Abbreviations: ASSIST, Alcohol Smoking and Substance Involvement Screening Test; ED, emergency department; hi, high; Mod, moderate.

## Supporting information


**Table S1.** Baseline predictors of being excluded from the study estimated using univariable modified Poisson log‐linked regression.
**Table S2.** Sensitivity analysis including all substance‐related hospitalisations occurring on the same day as separate failure events.
**Table S3.** Multivariable Weibull regression comparing hazard rates of opioid‐related hospitalisation between low/no and moderate/high risk heroin groups for 3 months after release from prison.
**Table S4.** Multivariable Weibull regression comparing hazard rates of methamphetamine‐related hospitalisation between low/no and moderate/high risk methamphetamine groups for 3 months after release from prison.
**Table S5.** Multivariable Weibull regression comparing hazard rates of cannabis‐related hospitalisation between low/no and moderate/high risk cannabis groups for 3 months after release from prison.
**Table S6.** Multivariable Weibull regression comparing hazard rates of opioid‐related hospitalisation between low/no and moderate/high risk heroin groups for 6 months after release from prison.
**Table S7.** Multivariable Weibull regression comparing hazard rates of methamphetamine‐related hospitalisation between low/no and moderate/high risk methamphetamine groups for 6 months after release from prison.
**Table S8.** Multivariable Weibull regression comparing hazard rates of cannabis‐related hospitalisation between low/no and moderate/high risk cannabis groups for 6 months after release from prison.
**Table S9.** Multivariable Weibull regression comparing hazard rates of opioid‐related hospitalisation between low/no and moderate/high risk heroin groups for 12 months after release from prison.
**Table S10.** Multivariable Weibull regression comparing hazard rates of methamphetamine‐related hospitalisation between low/no and moderate/high risk methamphetamine groups for 12 months after release from prison.
**Table S11.** Multivariable Weibull regression comparing hazard rates of cannabis‐related hospitalisation between low/no and moderate/high risk cannabis groups for 12 months after release from prison.
**Table S12.** Multivariable Weibull regression comparing hazard rates of opioid‐related hospitalisation between low/no and moderate/high risk heroin groups for entire follow‐up period.
**Table S13.** Multivariable Weibull regression comparing hazard rates of methamphetamine‐related hospitalisation between low/no and moderate/high risk methamphetamine groups for entire follow‐up period.
**Table S14.** Multivariable Weibull regression comparing hazard rates of cannabis‐related hospitalisation between low/no and moderate/high risk cannabis groups for entire follow‐up period.
**Table S15.** Multivariable Weibull regression comparing hazard rates of any‐substance‐related hospitalisation between low/no and moderate/high risk heroin groups for 3 months after release from prison.
**Table S16.** Multivariable Weibull regression comparing hazard rates of any‐substance‐related hospitalisation between low/no and moderate/high risk methamphetamine groups for 3 months after release from prison.
**Table S17.** Multivariable Weibull regression comparing hazard rates of any‐substance‐related hospitalisation between low/no and moderate/high risk cannabis groups for 3 months after release from prison.
**Table S18.** Multivariable Weibull regression comparing hazard rates of any‐substance‐related hospitalisation between low/no and moderate/high risk heroin groups for 6 months after release from prison.
**Table S19.** Multivariable Weibull regression comparing hazard rates of any‐substance‐related hospitalisation between low/no and moderate/high risk methamphetamine groups for 6 months after release from prison.
**Table S20.** Multivariable Weibull regression comparing hazard rates of any‐substance‐related hospitalisation between low/no and moderate/high risk cannabis groups for 6 months after release from prison.
**Table S21.** Multivariable Weibull regression comparing hazard rates of any‐substance‐related hospitalisation between low/no and moderate/high risk heroin groups for 12 months after release from prison.
**Table S22.** Multivariable Weibull regression comparing hazard rates of any‐substance‐related hospitalisation between low/no and moderate/high risk methamphetamine groups for 12 months after release from prison.
**Table S23.** Multivariable Weibull regression comparing hazard rates of any‐substance‐related hospitalisation between low/no and moderate/high risk cannabis groups for 12 months after release from prison.
**Table S24.** Multivariable Weibull regression comparing hazard rates of any‐substance‐related hospitalisation between low/no and moderate/high risk heroin groups for the entire follow‐up period.
**Table S25.** Multivariable Weibull regression comparing hazard rates of any‐substance‐related hospitalisation between low/no and moderate/high risk methamphetamine groups for the entire follow‐up period.
**Table S26.** Multivariable Weibull regression comparing hazard rates of any‐substance‐related hospitalisation between low/no and moderate/high risk cannabis groups for the entire follow‐up period.

## Data Availability

Research data are not shared.
