# Supplementary material for: Using the Alcohol, Smoking and Substance Involvement Screening Test to predict substance‐related hospitalisation after release from prison: A cohort study
Source: Addiction. 2023 Oct 19;119(2):236–47. doi: 10.1111/add.16365 (PMC10952305; doi:10.1111/add.16365)
Supplement: Supplementary file 1 — Table S1. Baseline predictors of being excluded from the study estimated using univariable modified Poisson log‐linked regression. Table S2. Sensitivity analysis including all substance‐related hospitalisations occurring on the same day as separate failure events. Table S3. Multivariable Weibull regression comparing hazard rates of opioid‐related hospitalisation between low/no and moderate/high risk heroin groups for 3 months after release from prison. Table S4. Multivariable Weibull regression comparing hazard rates of methamphetamine‐related hospitalisation between low/no and moderate/high risk methamphetamine groups for 3 months after release from prison. Table S5. Multivariable Weibull regression comparing hazard rates of cannabis‐related hospitalisation between low/no and moderate/high risk cannabis groups for 3 months after release from prison. Table S6. Multivariable Weibull regression comparing hazard rates of opioid‐related hospitalisation between low/no and moderate/high risk heroin groups for 6 months after release from prison. Table S7. Multivariable Weibull regression comparing hazard rates of methamphetamine‐related hospitalisation between low/no and moderate/high risk methamphetamine groups for 6 months after release from prison. Table S8. Multivariable Weibull regression comparing hazard rates of cannabis‐related hospitalisation between low/no and moderate/high risk cannabis groups for 6 months after release from prison. Table S9. Multivariable Weibull regression comparing hazard rates of opioid‐related hospitalisation between low/no and moderate/high risk heroin groups for 12 months after release from prison. Table S10. Multivariable Weibull regression comparing hazard rates of methamphetamine‐related hospitalisation between low/no and moderate/high risk methamphetamine groups for 12 months after release from prison. Table S11. Multivariable Weibull regression comparing hazard rates of cannabis‐related hospitalisation between low/no and moderate/hig [file ADD-119-236-s001.docx]

# Supplementary materials

## Covariates for multivariable models

Covariates for the multivariable Weibull Regression models were selected based on empirical evidence of an association with substance use and/or substance-related outcomes, such as overdose. More specifically, age and sex are both associated with injecting substance use and/or overdose, (1-4) and there is also evidence of elevated rates substance use, as well as overdose and related deaths among Indigenous populations compared to non-Indigenous populations.(5, 6) People with a history of injecting drug use are also at an increased risk of overdose compared to people with no injecting drug use history.(7) Among people leaving prison, length of preceding incarceration,(8, 9) being unemployed before the preceding incarceration,(8, 10) a history of being removed from family during childhood,(9, 10) perceived low social support assessed by the Enrichd Social Support Inventory (ESSI),(9, 11) previous diagnosis of mental illness,(10) experiencing high psychological distress prior to release from prison as measured by the Kessler Psychological Distress Scale (K10),(10, 12) and a history of non-fatal overdose (9) are all associated with overdose after leaving prison. Based on this evidence, these variables were included as covariates in the multivariable model.

### Selection bias analysis

We used items from the baseline survey to investigate possible selection bias. Sociodemographic and health variables included sex (male/female), Indigeneity (yes/no), age (<25/25+ years at baseline), employed in the six months prior to index incarceration (yes/no), taken from family as a child (yes/no), low/no vs moderate/high/very high levels of psychological distress at baseline as measured by the K10,(13) previous diagnosis of a mental illness (yes/no), a history of injecting drugs (yes/no), a history of drug overdose (yes/no), level of social support as measured by the ESSI (11) dichotomised at the median (<28/28+).(14) To investigate whether screening as moderate/high risk for the use of any of the three substances being investigated was associated with being excluded from the study, we generated a binary variable (moderate/high risk vs low/no risk) and assigned participants to the moderate/high risk group if they screen as moderate/high risk on the ASSIST at baseline for any of the three substances. For each variable we used univariable modified log-linked Poisson regression to estimate the relative risk of being excluded.

### Sensitivity analysis

We conducted sensitivity analysis to investigate our assumption that multiple specific-substance-related hospital contacts that occurred within 24 hours of each other for the same participant related to the same substance use event (resulting in us combining all hospital contacts occurring on the same day for the same individual into one event for our main analyses). To conduct the sensitivity analysis, we repeated our unadjusted and adjusted Weibull regression survival analyses across the entire follow-up period, however we left all hospital contact events as separate events to see how this impacted the unadjusted and adjusted hazard ratio estimates.

## Results

### Selection bias analysis

Table S1. Baseline predictors of being excluded from the study estimated using univariable modified Poisson log-linked regression

| Excluded from analysis | RR (95%CI) | P |
| --- | --- | --- |
| Female | 0.53 (0.30, 0.94) | 0.030 |
| Indigenous | 2.22 (1.54, 3.19) | <0.001 |
| < 25 years old | 1.20 (0.82, 1.76) | 0.335 |
| Employed 6 months pre-incarceration | 0.76 (0.52, 1.12) | 0.166 |
| Taken into care as a child | 0.66 (0.37, 1.17) | 0.157 |
| Moderate/high distress (K10) | 1.23 (0.80, 1.90) | 0.344 |
| Ever diagnosed with mental illness | 1.01 (0.66, 1.54) | 0.978 |
| Injected drugs ever | 0.68 (0.43, 1.07) | 0.095 |
| Previously overdosed | 1.33 (0.81, 2.17) | 0.259 |
| Low social support (ESSI < 25) | 1.09 (0.75, 1.58) | 0.658 |
| Moderate/high risk use of any substance (ASSIST) | 1.01 (0.61, 1.68) | 0.967 |

RR: relative risk; K10: 10-item Kessler Psychological Distress Scale; ESSI: Enrichd Social Support Inventory; ASSIST: Alcohol Smoking and Substance Involvement Screening Test

### Sensitivity analysis

Table S2. Sensitivity analysis including all substance-related hospitalisations occurring on the same day as separate failure events

|  | Cannabis-related | | Methamphetamine-related | | Opioid-related | | Any substance-related | |
| --- | --- | --- | --- | --- | --- | --- | --- | --- |
|  | HR (95%CI) | aHR^a^ (95%CI) | HR (95%CI) | aHR^a^ (9%CI) | HR (95%CI) | aHR^a^ (9%CI) | HR (95%CI) | aHR^a^ (9%CI) |
| Entire follow |  |  |  |  |  |  |  |  |
| Cannabis | 3.47 (2.59, 4.65) | 2.34 (1.72, 3.19) |  |  |  |  | 1.86 (1.71, 2.02) | 1.33 (1.22, 1.45) |
| Methamphetamine |  |  | 4.60 (3.73, 5.67) | 2.26 (1.78, 2.87) |  |  | 1.42 (1.32, 1.55) | 0.82 (0.75, 0.90) |
| Heroin |  |  |  |  | 12.25 (9.74, 15.40) | 5.55 (4.27, 7.21) | 1.63 (1.48, 1.80) | 1.12 (1.00, 1.24) |

## Full multivariable Poisson regression models

### Specific substance-related hospitalisation

Table S3. Multivariable Weibull regression comparing hazard rates of opioid-related hospitalisation between low/no and moderate/high risk heroin groups for 3 months after release from prison

| Opioid-related hospitalisation | aHR (95%CI) | P-value |
| --- | --- | --- |
| Moderate/high risk heroin use (ASSIST) | 19.95 (8.2, 48.54) | 0.000 |
| Indigenous | 1.54 (0.77, 3.08) | 0.218 |
| Female | 1.53 (0.75, 3.09) | 0.241 |
| Age (vs 1 year younger) | 1.01 (0.98, 1.05) | 0.443 |
| Employed in 6 months before prison | 0.67 (0.31, 1.46) | 0.313 |
| Index Sentence (ref > 365 days) |  |  |
| ≤90 days | 0.99 (0.43, 2.27) | 0.982 |
| 91-365 days | 0.55 (0.27, 1.14) | 0.109 |
| Taken from family as a child | 1.27 (0.63, 2.54) | 0.507 |
| Moderate/high distress (K10) | 1.03 (0.52, 2.02) | 0.941 |
| Mental illness previously diagnosed | 0.69 (0.35, 1.34) | 0.270 |
| Ever injected drugs* | - | - |
| Ever overdosed | 1.83 (0.91, 3.67) | 0.090 |
| Low social support (ESSI < 28) | 2.61 (1.3, 5.27) | 0.007 |

aHR: adjusted hazard ratio; ASSIST: Alcohol Smoking and Substance Involvement Screening Test; K10: 10-item Kessler Psychological Distress Scale; ESSI: Enrichd Social Support Inventory

Table S4. Multivariable Weibull regression comparing hazard rates of methamphetamine-related hospitalisation between low/no and moderate/high risk methamphetamine groups for 3 months after release from prison

| Methamphetamine-related hospitalisation | aHR (95%CI) | P-value |
| --- | --- | --- |
| Moderate/high risk methamphetamine use (ASSIST) | 3.14 (1.24, 7.95) | 0.016 |
| Indigenous | 1.95 (1.05, 3.61) | 0.034 |
| Female | 1.38 (0.71, 2.68) | 0.348 |
| Age (vs 1 year younger) | 0.99 (0.95, 1.03) | 0.528 |
| Employed in 6 months before prison | 0.79 (0.39, 1.61) | 0.511 |
| Index Sentence (ref > 365 days) |  |  |
| ≤90 days | 0.69 (0.26, 1.81) | 0.450 |
| 91-365 days | 0.9 (0.48, 1.69) | 0.744 |
| Taken from family as a child | 0.54 (0.23, 1.3) | 0.170 |
| Moderate/high distress (K10) | 0.88 (0.48, 1.62) | 0.688 |
| Mental illness previously diagnosed | 1.68 (0.87, 3.23) | 0.122 |
| Ever injected drugs | 4.3 (1.21, 15.31) | 0.024 |
| Ever overdosed | 1.39 (0.76, 2.57) | 0.287 |
| Low social support (ESSI < 28) | 1.18 (0.66, 2.12) | 0.578 |

aHR: adjusted hazard ratio; ASSIST: Alcohol Smoking and Substance Involvement Screening Test; K10: 10-item Kessler Psychological Distress Scale; ESSI: Enrichd Social Support Inventory

Table S5. Multivariable Weibull regression comparing hazard rates of cannabis-related hospitalisation between low/no and moderate/high risk cannabis groups for 3 months after release from prison

| Cannabis-related hospitalisation | aHR (95%CI) | P-value |
| --- | --- | --- |
| Moderate/high risk cannabis use (ASSIST) | 3.72 (0.83, 16.7) | 0.087 |
| Indigenous | 1.46 (0.5, 4.28) | 0.485 |
| Female | 1.38 (0.44, 4.35) | 0.581 |
| Age (vs 1 year younger) | 0.97 (0.91, 1.04) | 0.423 |
| Employed in 6 months before prison | 0.17 (0.02, 1.37) | 0.096 |
| Index Sentence (ref > 365 days) |  |  |
| ≤90 days | 1.31 (0.32, 5.31) | 0.703 |
| 91-365 days | 0.87 (0.26, 2.9) | 0.814 |
| Taken from family as a child | 1.12 (0.34, 3.65) | 0.856 |
| Moderate/high distress (K10) | 1.74 (0.52, 5.81) | 0.365 |
| Mental illness previously diagnosed | 1.02 (0.33, 3.13) | 0.975 |
| Ever injected drugs | 1.37 (0.36, 5.17) | 0.642 |
| Ever overdosed | 3.73 (1.26, 11.03) | 0.017 |
| Low social support (ESSI < 28) | 0.85 (0.3, 2.43) | 0.765 |

aHR: adjusted hazard ratio; ASSIST: Alcohol Smoking and Substance Involvement Screening Test; K10: 10-item Kessler Psychological Distress Scale; ESSI: Enrichd Social Support Inventory

Table S6. Multivariable Weibull regression comparing hazard rates of opioid-related hospitalisation between low/no and moderate/high risk heroin groups for 6 months after release from prison

| Opioid-related hospitalisation | aHR (95%CI) | P-value |
| --- | --- | --- |
| Moderate/high risk heroin use (ASSIST) | 8.15 (4.5, 14.75) | 0.000 |
| Indigenous | 2.3 (1.39, 3.81) | 0.001 |
| Female | 1.5 (0.9, 2.51) | 0.119 |
| Age (vs 1 year younger) | 1.03 (1, 1.06) | 0.035 |
| Employed in 6 months before prison | 0.54 (0.28, 1.04) | 0.065 |
| Index Sentence (ref > 365 days) |  |  |
| ≤90 days | 1.06 (0.54, 2.06) | 0.869 |
| 91-365 days | 0.78 (0.46, 1.33) | 0.364 |
| Taken from family as a child | 1.02 (0.6, 1.76) | 0.934 |
| Moderate/high distress (K10) | 1 (0.6, 1.67) | 0.998 |
| Mental illness previously diagnosed | 1.02 (0.6, 1.71) | 0.955 |
| Ever injected drugs | 12.85 (1.7, 97.15) | 0.013 |
| Ever overdosed | 2.05 (1.2, 3.48) | 0.008 |
| Low social support (ESSI < 28) | 2.51 (1.46, 4.3) | 0.001 |

aHR: adjusted hazard ratio; ASSIST: Alcohol Smoking and Substance Involvement Screening Test; K10: 10-item Kessler Psychological Distress Scale; ESSI: Enrichd Social Support Inventory

Table S7. Multivariable Weibull regression comparing hazard rates of methamphetamine-related hospitalisation between low/no and moderate/high risk methamphetamine groups for 6 months after release from prison

| Methamphetamine-related hospitalisation | aHR (95%CI) | P-value |
| --- | --- | --- |
| Moderate/high risk methamphetamine use (ASSIST) | 3.08 (1.61, 5.87) | 0.001 |
| Indigenous | 1.86 (1.19, 2.9) | 0.007 |
| Female | 1.15 (0.7, 1.88) | 0.585 |
| Age (vs 1 year younger) | 1 (0.97, 1.02) | 0.798 |
| Employed in 6 months before prison | 0.92 (0.56, 1.5) | 0.739 |
| Index Sentence (ref > 365 days) |  |  |
| ≤90 days | 0.6 (0.29, 1.24) | 0.165 |
| 91-365 days | 0.89 (0.56, 1.4) | 0.606 |
| Taken from family as a child | 0.88 (0.52, 1.52) | 0.655 |
| Moderate/high distress (K10) | 0.91 (0.58, 1.41) | 0.660 |
| Mental illness previously diagnosed | 2.02 (1.25, 3.28) | 0.004 |
| Ever injected drugs | 3.69 (1.58, 8.61) | 0.003 |
| Ever overdosed | 1.07 (0.68, 1.68) | 0.761 |
| Low social support (ESSI < 28) | 1.39 (0.91, 2.12) | 0.131 |

aHR: adjusted hazard ratio; ASSIST: Alcohol Smoking and Substance Involvement Screening Test; K10: 10-item Kessler Psychological Distress Scale; ESSI: Enrichd Social Support Inventory

Table S8. Multivariable Weibull regression comparing hazard rates of cannabis-related hospitalisation between low/no and moderate/high risk cannabis groups for 6 months after release from prison

| Cannabis-related hospitalisation | aHR (95%CI) | P-value |
| --- | --- | --- |
| Moderate/high risk cannabis use (ASSIST) | 2.99 (1.3, 6.88) | 0.010 |
| Indigenous | 1.43 (0.74, 2.76) | 0.294 |
| Female | 1.61 (0.79, 3.27) | 0.187 |
| Age (vs 1 year younger) | 0.99 (0.95, 1.03) | 0.508 |
| Employed in 6 months before prison | 0.38 (0.15, 0.93) | 0.034 |
| Index Sentence (ref > 365 days) |  |  |
| ≤90 days | 0.66 (0.24, 1.76) | 0.405 |
| 91-365 days | 0.8 (0.41, 1.59) | 0.530 |
| Taken from family as a child | 0.69 (0.3, 1.58) | 0.377 |
| Moderate/high distress (K10) | 1.09 (0.56, 2.13) | 0.794 |
| Mental illness previously diagnosed | 1.53 (0.75, 3.12) | 0.240 |
| Ever injected drugs | 1.36 (0.62, 2.98) | 0.436 |
| Ever overdosed | 1.78 (0.92, 3.44) | 0.085 |
| Low social support (ESSI < 28) | 1.76 (0.92, 3.37) | 0.087 |

aHR: adjusted hazard ratio; ASSIST: Alcohol Smoking and Substance Involvement Screening Test; K10: 10-item Kessler Psychological Distress Scale; ESSI: Enrichd Social Support Inventory

Table S9. Multivariable Weibull regression comparing hazard rates of opioid-related hospitalisation between low/no and moderate/high risk heroin groups for 12 months after release from prison

| Opioid-related hospitalisation | aHR (95%CI) | P-value |
| --- | --- | --- |
| Moderate/high risk heroin use (ASSIST) | 6.33 (4.09, 9.79) | 0.000 |
| Indigenous | 1.62 (1.08, 2.42) | 0.019 |
| Female | 1.37 (0.92, 2.04) | 0.122 |
| Age (vs 1 year younger) | 1.02 (1, 1.05) | 0.026 |
| Employed in 6 months before prison | 0.85 (0.55, 1.31) | 0.451 |
| Index Sentence (ref > 365 days) |  |  |
| ≤90 days | 1.42 (0.86, 2.35) | 0.173 |
| 91-365 days | 1 (0.65, 1.53) | 0.998 |
| Taken from family as a child | 0.87 (0.56, 1.35) | 0.522 |
| Moderate/high distress (K10) | 1.1 (0.74, 1.64) | 0.632 |
| Mental illness previously diagnosed | 1.28 (0.85, 1.93) | 0.246 |
| Ever injected drugs | 8.89 (2.71, 29.22) | 0.000 |
| Ever overdosed | 1.9 (1.27, 2.86) | 0.002 |
| Low social support (ESSI < 28) | 1.91 (1.28, 2.85) | 0.002 |

aHR: adjusted hazard ratio; ASSIST: Alcohol Smoking and Substance Involvement Screening Test; K10: 10-item Kessler Psychological Distress Scale; ESSI: Enrichd Social Support Inventory

Table S10. Multivariable Weibull regression comparing hazard rates of methamphetamine-related hospitalisation between low/no and moderate/high risk methamphetamine groups for 12 months after release from prison

| Methamphetamine-related hospitalisation | aHR (95%CI) | P-value |
| --- | --- | --- |
| Moderate/high risk methamphetamine use (ASSIST) | 3 (1.93, 4.66) | 0.000 |
| Indigenous | 1.78 (1.29, 2.43) | 0.000 |
| Female | 1.1 (0.78, 1.55) | 0.595 |
| Age (vs 1 year younger) | 0.99 (0.98, 1.01) | 0.529 |
| Employed in 6 months before prison | 0.81 (0.57, 1.16) | 0.246 |
| Index Sentence (ref > 365 days) |  |  |
| ≤90 days | 0.61 (0.37, 1.01) | 0.056 |
| 91-365 days | 0.9 (0.65, 1.25) | 0.546 |
| Taken from family as a child | 1.28 (0.9, 1.82) | 0.168 |
| Moderate/high distress (K10) | 1.36 (0.98, 1.9) | 0.066 |
| Mental illness previously diagnosed | 1.82 (1.29, 2.55) | 0.001 |
| Ever injected drugs | 2.56 (1.51, 4.33) | 0.000 |
| Ever overdosed | 0.93 (0.67, 1.29) | 0.652 |
| Low social support (ESSI < 28) | 1.09 (0.81, 1.47) | 0.586 |

aHR: adjusted hazard ratio; ASSIST: Alcohol Smoking and Substance Involvement Screening Test; K10: 10-item Kessler Psychological Distress Scale; ESSI: Enrichd Social Support Inventory

Table S11. Multivariable Weibull regression comparing hazard rates of cannabis-related hospitalisation between low/no and moderate/high risk cannabis groups for 12 months after release from prison

| Cannabis-related hospitalisation | aHR (95%CI) | P-value |
| --- | --- | --- |
| Moderate/high risk cannabis use (ASSIST) | 2.98 (1.7, 5.2) | 0.000 |
| Indigenous | 1.5 (0.95, 2.35) | 0.080 |
| Female | 1.22 (0.75, 1.98) | 0.430 |
| Age (vs 1 year younger) | 1 (0.98, 1.03) | 0.956 |
| Employed in 6 months before prison | 0.64 (0.38, 1.08) | 0.093 |
| Index Sentence (ref > 365 days) |  |  |
| ≤90 days | 0.82 (0.42, 1.6) | 0.563 |
| 91-365 days | 0.98 (0.61, 1.57) | 0.927 |
| Taken from family as a child | 0.8 (0.47, 1.38) | 0.423 |
| Moderate/high distress (K10) | 1.3 (0.82, 2.05) | 0.268 |
| Mental illness previously diagnosed | 1.82 (1.12, 2.95) | 0.016 |
| Ever injected drugs | 2.14 (1.21, 3.78) | 0.008 |
| Ever overdosed | 1.03 (0.65, 1.64) | 0.898 |
| Low social support (ESSI < 28) | 1.54 (1, 2.37) | 0.051 |

aHR: adjusted hazard ratio; ASSIST: Alcohol Smoking and Substance Involvement Screening Test; K10: 10-item Kessler Psychological Distress Scale; ESSI: Enrichd Social Support Inventory

Table S12. Multivariable Weibull regression comparing hazard rates of opioid-related hospitalisation between low/no and moderate/high risk heroin groups for entire follow-up period

| Opioid-related hospitalisation | aHR (95%CI) | P-value |
| --- | --- | --- |
| Moderate/high risk heroin use (ASSIST) | 5.79 (4.41, 7.6) | 0.000 |
| Indigenous | 1.15 (0.89, 1.5) | 0.290 |
| Female | 1.78 (1.39, 2.28) | 0.000 |
| Age (vs 1 year younger) | 1.02 (1.01, 1.03) | 0.004 |
| Employed in 6 months before prison | 0.83 (0.63, 1.09) | 0.180 |
| Index Sentence (ref > 365 days) |  |  |
| ≤90 days | 0.98 (0.7, 1.38) | 0.898 |
| 91-365 days | 1.24 (0.94, 1.62) | 0.122 |
| Taken from family as a child | 0.86 (0.64, 1.14) | 0.297 |
| Moderate/high distress (K10) | 1.14 (0.89, 1.47) | 0.289 |
| Mental illness previously diagnosed | 1.47 (1.13, 1.91) | 0.005 |
| Ever injected drugs | 4.1 (2.45, 6.87) | 0.000 |
| Ever overdosed | 1.47 (1.14, 1.9) | 0.003 |
| Low social support (ESSI < 28) | 1.44 (1.13, 1.84) | 0.003 |

aHR: adjusted hazard ratio; ASSIST: Alcohol Smoking and Substance Involvement Screening Test; K10: 10-item Kessler Psychological Distress Scale; ESSI: Enrichd Social Support Inventory

Table S13. Multivariable Weibull regression comparing hazard rates of methamphetamine-related hospitalisation between low/no and moderate/high risk methamphetamine groups for entire follow-up period

| Methamphetamine-related hospitalisation | aHR (95%CI) | P-value |
| --- | --- | --- |
| Moderate/high risk methamphetamine use (ASSIST) | 2.23 (1.75, 2.84) | 0.000 |
| Indigenous | 2.05 (1.7, 2.48) | 0.000 |
| Female | 1.04 (0.85, 1.27) | 0.707 |
| Age (vs 1 year younger) | 0.99 (0.97, 1) | 0.011 |
| Employed in 6 months before prison | 0.59 (0.47, 0.74) | 0.000 |
| Index Sentence (ref > 365 days) |  |  |
| ≤90 days | 0.48 (0.35, 0.65) | 0.000 |
| 91-365 days | 0.93 (0.77, 1.13) | 0.464 |
| Taken from family as a child | 1.22 (0.99, 1.5) | 0.066 |
| Moderate/high distress (K10) | 1.31 (1.08, 1.58) | 0.006 |
| Mental illness previously diagnosed | 1.25 (1.03, 1.52) | 0.022 |
| Ever injected drugs | 3.13 (2.3, 4.25) | 0.000 |
| Ever overdosed | 1.02 (0.84, 1.24) | 0.846 |
| Low social support (ESSI < 28) | 1 (0.84, 1.2) | 0.957 |

aHR: adjusted hazard ratio; ASSIST: Alcohol Smoking and Substance Involvement Screening Test; K10: 10-item Kessler Psychological Distress Scale; ESSI: Enrichd Social Support Inventory

Table S14. Multivariable Weibull regression comparing hazard rates of cannabis-related hospitalisation between low/no and moderate/high risk cannabis groups for entire follow-up period

| Cannabis-related hospitalisation | aHR (95%CI) | P-value |
| --- | --- | --- |
| Moderate/high risk cannabis use (ASSIST) | 2.38 (1.74, 3.24) | 0.000 |
| Indigenous | 1.76 (1.35, 2.28) | 0.000 |
| Female | 1.14 (0.85, 1.52) | 0.373 |
| Age (vs 1 year younger) | 0.99 (0.97, 1) | 0.089 |
| Employed in 6 months before prison | 0.54 (0.4, 0.74) | 0.000 |
| Index Sentence (ref > 365 days) |  |  |
| ≤90 days | 0.5 (0.33, 0.76) | 0.001 |
| 91-365 days | 0.84 (0.65, 1.11) | 0.220 |
| Taken from family as a child | 1.01 (0.75, 1.37) | 0.935 |
| Moderate/high distress (K10) | 1.27 (0.98, 1.65) | 0.073 |
| Mental illness previously diagnosed | 1.42 (1.08, 1.86) | 0.011 |
| Ever injected drugs | 1.54 (1.15, 2.07) | 0.004 |
| Ever overdosed | 1.11 (0.85, 1.46) | 0.435 |
| Low social support (ESSI < 28) | 1.01 (0.79, 1.29) | 0.963 |

aHR: adjusted hazard ratio; ASSIST: Alcohol Smoking and Substance Involvement Screening Test; K10: 10-item Kessler Psychological Distress Scale; ESSI: Enrichd Social Support Inventory

### Any-substance related hospitalisation

Table S15. Multivariable Weibull regression comparing hazard rates of any-substance-related hospitalisation between low/no and moderate/high risk heroin groups for 3 months after release from prison

| Any-substance-related hospitalisation | aHR (95%CI) | P-value |
| --- | --- | --- |
| Moderate/high risk heroin use (ASSIST) | 1.53 (1.11, 2.11) | 0.010 |
| Indigenous | 2.14 (1.61, 2.84) | 0.000 |
| Female | 1.03 (0.76, 1.39) | 0.873 |
| Age (vs 1 year younger) | 1 (0.99, 1.02) | 0.914 |
| Employed in 6 months before prison | 0.5 (0.35, 0.71) | 0.000 |
| Index Sentence (ref > 365 days) |  |  |
| ≤90 days | 1.07 (0.72, 1.57) | 0.747 |
| 91-365 days | 0.91 (0.68, 1.22) | 0.535 |
| Taken from family as a child | 1.05 (0.77, 1.44) | 0.736 |
| Moderate/high distress (K10) | 1.03 (0.78, 1.37) | 0.815 |
| Mental illness previously diagnosed | 2.03 (1.49, 2.75) | 0.000 |
| Ever injected drugs | 1.65 (1.17, 2.34) | 0.005 |
| Ever overdosed | 1.31 (0.98, 1.76) | 0.070 |
| Low social support (ESSI < 28) | 1.79 (1.36, 2.37) | 0.000 |

aHR: adjusted hazard ratio; ASSIST: Alcohol Smoking and Substance Involvement Screening Test; K10: 10-item Kessler Psychological Distress Scale; ESSI: Enrichd Social Support Inventory

Table S16. Multivariable Weibull regression comparing hazard rates of any-substance-related hospitalisation between low/no and moderate/high risk methamphetamine groups for 3 months after release from prison

| Any-substance-related hospitalisation | aHR (95%CI) | P-value |
| --- | --- | --- |
| Moderate/high risk methamphetamine use (ASSIST) | 0.6 (0.45, 0.81) | 0.001 |
| Indigenous | 1.95 (1.47, 2.58) | 0.000 |
| Female | 1.07 (0.79, 1.45) | 0.656 |
| Age (vs 1 year younger) | 1 (0.98, 1.01) | 0.903 |
| Employed in 6 months before prison | 0.47 (0.33, 0.67) | 0.000 |
| Index Sentence (ref > 365 days) |  |  |
| ≤90 days | 0.97 (0.66, 1.43) | 0.877 |
| 91-365 days | 0.87 (0.65, 1.17) | 0.363 |
| Taken from family as a child | 1.04 (0.76, 1.41) | 0.827 |
| Moderate/high distress (K10) | 1.03 (0.78, 1.37) | 0.819 |
| Mental illness previously diagnosed | 2.03 (1.49, 2.75) | 0.000 |
| Ever injected drugs | 2.34 (1.63, 3.37) | 0.000 |
| Ever overdosed | 1.52 (1.14, 2.01) | 0.004 |
| Low social support (ESSI < 28) | 1.79 (1.36, 2.36) | 0.000 |

aHR: adjusted hazard ratio; ASSIST: Alcohol Smoking and Substance Involvement Screening Test; K10: 10-item Kessler Psychological Distress Scale; ESSI: Enrichd Social Support Inventory

Table S17. Multivariable Weibull regression comparing hazard rates of any-substance-related hospitalisation between low/no and moderate/high risk cannabis groups for 3 months after release from prison

| Any-substance-related hospitalisation | aHR (95%CI) | P-value |
| --- | --- | --- |
| Moderate/high risk cannabis use (ASSIST) | 0.97 (0.74, 1.28) | 0.842 |
| Indigenous | 2.02 (1.53, 2.68) | 0.000 |
| Female | 1.04 (0.77, 1.41) | 0.788 |
| Age (vs 1 year younger) | 1 (0.99, 1.02) | 0.855 |
| Employed in 6 months before prison | 0.49 (0.35, 0.69) | 0.000 |
| Index Sentence (ref > 365 days) |  |  |
| ≤90 days | 1.03 (0.7, 1.52) | 0.862 |
| 91-365 days | 0.89 (0.66, 1.19) | 0.419 |
| Taken from family as a child | 1.07 (0.78, 1.45) | 0.677 |
| Moderate/high distress (K10) | 1.03 (0.77, 1.37) | 0.847 |
| Mental illness previously diagnosed | 2.04 (1.5, 2.78) | 0.000 |
| Ever injected drugs | 1.82 (1.3, 2.55) | 0.001 |
| Ever overdosed | 1.45 (1.09, 1.92) | 0.010 |
| Low social support (ESSI < 28) | 1.79 (1.36, 2.36) | 0.000 |

aHR: adjusted hazard ratio; ASSIST: Alcohol Smoking and Substance Involvement Screening Test; K10: 10-item Kessler Psychological Distress Scale; ESSI: Enrichd Social Support Inventory

Table S18. Multivariable Weibull regression comparing hazard rates of any-substance-related hospitalisation between low/no and moderate/high risk heroin groups for 6 months after release from prison

| Any-substance-related hospitalisation | aHR (95%CI) | P-value |
| --- | --- | --- |
| Moderate/high risk heroin use (ASSIST) | 1.32 (1.04, 1.67) | 0.022 |
| Indigenous | 1.99 (1.63, 2.44) | 0.000 |
| Female | 1.12 (0.91, 1.39) | 0.289 |
| Age (vs 1 year younger) | 1.01 (1, 1.02) | 0.109 |
| Employed in 6 months before prison | 0.5 (0.39, 0.64) | 0.000 |
| Index Sentence (ref > 365 days) |  |  |
| ≤90 days | 0.93 (0.69, 1.23) | 0.599 |
| 91-365 days | 0.97 (0.79, 1.2) | 0.786 |
| Taken from family as a child | 0.98 (0.78, 1.22) | 0.828 |
| Moderate/high distress (K10) | 1.03 (0.84, 1.26) | 0.790 |
| Mental illness previously diagnosed | 1.7 (1.37, 2.11) | 0.000 |
| Ever injected drugs | 1.79 (1.39, 2.29) | 0.000 |
| Ever overdosed | 1.41 (1.14, 1.73) | 0.001 |
| Low social support (ESSI < 28) | 1.81 (1.48, 2.21) | 0.000 |

aHR: adjusted hazard ratio; ASSIST: Alcohol Smoking and Substance Involvement Screening Test; K10: 10-item Kessler Psychological Distress Scale; ESSI: Enrichd Social Support Inventory

Table S19. Multivariable Weibull regression comparing hazard rates of any-substance-related hospitalisation between low/no and moderate/high risk methamphetamine groups for 6 months after release from prison

| Any-substance-related hospitalisation | aHR (95%CI) | P-value |
| --- | --- | --- |
| Moderate/high risk methamphetamine use (ASSIST) | 0.75 (0.6, 0.93) | 0.008 |
| Indigenous | 1.89 (1.54, 2.31) | 0.000 |
| Female | 1.15 (0.93, 1.43) | 0.194 |
| Age (vs 1 year younger) | 1.01 (1, 1.02) | 0.137 |
| Employed in 6 months before prison | 0.48 (0.38, 0.62) | 0.000 |
| Index Sentence (ref > 365 days) |  |  |
| ≤90 days | 0.88 (0.66, 1.17) | 0.383 |
| 91-365 days | 0.94 (0.76, 1.17) | 0.596 |
| Taken from family as a child | 0.96 (0.76, 1.21) | 0.726 |
| Moderate/high distress (K10) | 1.03 (0.84, 1.26) | 0.807 |
| Mental illness previously diagnosed | 1.69 (1.36, 2.1) | 0.000 |
| Ever injected drugs | 2.2 (1.69, 2.86) | 0.000 |
| Ever overdosed | 1.55 (1.27, 1.9) | 0.000 |
| Low social support (ESSI < 28) | 1.8 (1.48, 2.2) | 0.000 |

aHR: adjusted hazard ratio; ASSIST: Alcohol Smoking and Substance Involvement Screening Test; K10: 10-item Kessler Psychological Distress Scale; ESSI: Enrichd Social Support Inventory

Table S20. Multivariable Weibull regression comparing hazard rates of any-substance-related hospitalisation between low/no and moderate/high risk cannabis groups for 6 months after release from prison

| Any-substance-related hospitalisation | aHR (95%CI) | P-value |
| --- | --- | --- |
| Moderate/high risk cannabis use (ASSIST) | 1.23 (1.01, 1.51) | 0.043 |
| Indigenous | 1.89 (1.55, 2.31) | 0.000 |
| Female | 1.15 (0.93, 1.43) | 0.209 |
| Age (vs 1 year younger) | 1.01 (1, 1.02) | 0.045 |
| Employed in 6 months before prison | 0.5 (0.39, 0.64) | 0.000 |
| Index Sentence (ref > 365 days) |  |  |
| ≤90 days | 0.92 (0.69, 1.23) | 0.594 |
| 91-365 days | 0.97 (0.78, 1.19) | 0.745 |
| Taken from family as a child | 0.98 (0.79, 1.24) | 0.895 |
| Moderate/high distress (K10) | 1.03 (0.84, 1.26) | 0.810 |
| Mental illness previously diagnosed | 1.67 (1.34, 2.07) | 0.000 |
| Ever injected drugs | 1.83 (1.44, 2.33) | 0.000 |
| Ever overdosed | 1.49 (1.22, 1.83) | 0.000 |
| Low social support (ESSI < 28) | 1.8 (1.48, 2.2) | 0.000 |

aHR: adjusted hazard ratio; ASSIST: Alcohol Smoking and Substance Involvement Screening Test; K10: 10-item Kessler Psychological Distress Scale; ESSI: Enrichd Social Support Inventory

Table S21. Multivariable Weibull regression comparing hazard rates of any-substance-related hospitalisation between low/no and moderate/high risk heroin groups for 12 months after release from prison

| Any-substance-related hospitalisation | aHR (95%CI) | P-value |
| --- | --- | --- |
| Moderate/high risk heroin use (ASSIST) | 1.28 (1.07, 1.54) | 0.008 |
| Indigenous | 2.12 (1.83, 2.47) | 0.000 |
| Female | 1.05 (0.9, 1.24) | 0.521 |
| Age (vs 1 year younger) | 1.01 (1, 1.01) | 0.098 |
| Employed in 6 months before prison | 0.59 (0.49, 0.7) | 0.000 |
| Index Sentence (ref > 365 days) |  |  |
| ≤90 days | 0.86 (0.7, 1.08) | 0.192 |
| 91-365 days | 0.99 (0.85, 1.16) | 0.905 |
| Taken from family as a child | 1.02 (0.86, 1.21) | 0.789 |
| Moderate/high distress (K10) | 1.23 (1.06, 1.43) | 0.007 |
| Mental illness previously diagnosed | 1.64 (1.4, 1.91) | 0.000 |
| Ever injected drugs | 1.69 (1.42, 2.02) | 0.000 |
| Ever overdosed | 1.26 (1.08, 1.48) | 0.004 |
| Low social support (ESSI < 28) | 1.22 (1.06, 1.4) | 0.006 |

aHR: adjusted hazard ratio; ASSIST: Alcohol Smoking and Substance Involvement Screening Test; K10: 10-item Kessler Psychological Distress Scale; ESSI: Enrichd Social Support Inventory

Table S22. Multivariable Weibull regression comparing hazard rates of any-substance-related hospitalisation between low/no and moderate/high risk methamphetamine groups for 12 months after release from prison

| Any-substance-related hospitalisation | aHR (95%CI) | P-value |
| --- | --- | --- |
| Moderate/high risk methamphetamine use (ASSIST) | 0.82 (0.7, 0.96) | 0.015 |
| Indigenous | 2.03 (1.74, 2.36) | 0.000 |
| Female | 1.06 (0.9, 1.25) | 0.501 |
| Age (vs 1 year younger) | 1.01 (1, 1.01) | 0.105 |
| Employed in 6 months before prison | 0.57 (0.48, 0.68) | 0.000 |
| Index Sentence (ref > 365 days) |  |  |
| ≤90 days | 0.84 (0.68, 1.05) | 0.124 |
| 91-365 days | 0.96 (0.82, 1.12) | 0.598 |
| Taken from family as a child | 0.99 (0.83, 1.17) | 0.870 |
| Moderate/high distress (K10) | 1.23 (1.06, 1.43) | 0.008 |
| Mental illness previously diagnosed | 1.62 (1.39, 1.9) | 0.000 |
| Ever injected drugs | 1.96 (1.62, 2.37) | 0.000 |
| Ever overdosed | 1.39 (1.19, 1.62) | 0.000 |
| Low social support (ESSI < 28) | 1.21 (1.05, 1.39) | 0.009 |

aHR: adjusted hazard ratio; ASSIST: Alcohol Smoking and Substance Involvement Screening Test; K10: 10-item Kessler Psychological Distress Scale; ESSI: Enrichd Social Support Inventory

Table S23. Multivariable Weibull regression comparing hazard rates of any-substance-related hospitalisation between low/no and moderate/high risk cannabis groups for 12 months after release from prison

| Any-substance-related hospitalisation | aHR (95%CI) | P-value |
| --- | --- | --- |
| Moderate/high risk cannabis use (ASSIST) | 1.39 (1.19, 1.62) | 0.000 |
| Indigenous | 2.01 (1.73, 2.34) | 0.000 |
| Female | 1.08 (0.92, 1.27) | 0.354 |
| Age (vs 1 year younger) | 1.01 (1, 1.02) | 0.015 |
| Employed in 6 months before prison | 0.59 (0.5, 0.71) | 0.000 |
| Index Sentence (ref > 365 days) |  |  |
| ≤90 days | 0.88 (0.7, 1.09) | 0.237 |
| 91-365 days | 0.99 (0.85, 1.16) | 0.938 |
| Taken from family as a child | 1.03 (0.87, 1.22) | 0.744 |
| Moderate/high distress (K10) | 1.23 (1.06, 1.43) | 0.008 |
| Mental illness previously diagnosed | 1.59 (1.36, 1.86) | 0.000 |
| Ever injected drugs | 1.68 (1.42, 2) | 0.000 |
| Ever overdosed | 1.33 (1.14, 1.55) | 0.000 |
| Low social support (ESSI < 28) | 1.21 (1.05, 1.4) | 0.008 |

aHR: adjusted hazard ratio; ASSIST: Alcohol Smoking and Substance Involvement Screening Test; K10: 10-item Kessler Psychological Distress Scale; ESSI: Enrichd Social Support Inventory

Table S24. Multivariable Weibull regression comparing hazard rates of any-substance-related hospitalisation between low/no and moderate/high risk heroin groups for the entire follow-up period

| Any-substance-related hospitalisation | aHR (95%CI) | P-value |
| --- | --- | --- |
| Moderate/high risk heroin use (ASSIST) | 1.12 (0.99, 1.25) | 0.061 |
| Indigenous | 2.2 (2.01, 2.41) | 0.000 |
| Female | 1.16 (1.05, 1.27) | 0.003 |
| Age (vs 1 year younger) | 1 (1, 1.01) | 0.098 |
| Employed in 6 months before prison | 0.52 (0.47, 0.58) | 0.000 |
| Index Sentence (ref > 365 days) |  |  |
| ≤90 days | 0.81 (0.71, 0.93) | 0.003 |
| 91-365 days | 1.17 (1.06, 1.29) | 0.002 |
| Taken from family as a child | 1.1 (1, 1.22) | 0.054 |
| Moderate/high distress (K10) | 1.17 (1.07, 1.28) | 0.001 |
| Mental illness previously diagnosed | 1.3 (1.19, 1.43) | 0.000 |
| Ever injected drugs | 1.73 (1.56, 1.92) | 0.000 |
| Ever overdosed | 1.37 (1.25, 1.51) | 0.000 |
| Low social support (ESSI < 28) | 1.18 (1.08, 1.29) | 0.000 |

aHR: adjusted hazard ratio; ASSIST: Alcohol Smoking and Substance Involvement Screening Test; K10: 10-item Kessler Psychological Distress Scale; ESSI: Enrichd Social Support Inventory

Table S25. Multivariable Weibull regression comparing hazard rates of any-substance-related hospitalisation between low/no and moderate/high risk methamphetamine groups for the entire follow-up period

| Any-substance-related hospitalisation | aHR (95%CI) | P-value |
| --- | --- | --- |
| Moderate/high risk methamphetamine use (ASSIST) | 0.82 (0.74, 0.9) | 0.000 |
| Indigenous | 2.13 (1.94, 2.33) | 0.000 |
| Female | 1.14 (1.04, 1.26) | 0.007 |
| Age (vs 1 year younger) | 1 (1, 1.01) | 0.123 |
| Employed in 6 months before prison | 0.52 (0.46, 0.57) | 0.000 |
| Index Sentence (ref > 365 days) |  |  |
| ≤90 days | 0.81 (0.7, 0.93) | 0.002 |
| 91-365 days | 1.14 (1.04, 1.26) | 0.008 |
| Taken from family as a child | 1.05 (0.95, 1.17) | 0.312 |
| Moderate/high distress (K10) | 1.18 (1.07, 1.29) | 0.000 |
| Mental illness previously diagnosed | 1.29 (1.18, 1.42) | 0.000 |
| Ever injected drugs | 1.94 (1.73, 2.18) | 0.000 |
| Ever overdosed | 1.47 (1.34, 1.61) | 0.000 |
| Low social support (ESSI < 28) | 1.16 (1.07, 1.27) | 0.001 |

aHR: adjusted hazard ratio; ASSIST: Alcohol Smoking and Substance Involvement Screening Test; K10: 10-item Kessler Psychological Distress Scale; ESSI: Enrichd Social Support Inventory

Table S26. Multivariable Weibull regression comparing hazard rates of any-substance-related hospitalisation between low/no and moderate/high risk cannabis groups for the entire follow-up period

| Any-substance-related hospitalisation | aHR (95%CI) | P-value |
| --- | --- | --- |
| Moderate/high risk cannabis use (ASSIST) | 1.35 (1.23, 1.48) | 0.000 |
| Indigenous | 2.12 (1.93, 2.32) | 0.000 |
| Female | 1.18 (1.07, 1.3) | 0.001 |
| Age (vs 1 year younger) | 1.01 (1, 1.01) | 0.006 |
| Employed in 6 months before prison | 0.53 (0.48, 0.59) | 0.000 |
| Index Sentence (ref > 365 days) |  |  |
| ≤90 days | 0.83 (0.73, 0.95) | 0.009 |
| 91-365 days | 1.18 (1.07, 1.3) | 0.001 |
| Taken from family as a child | 1.11 (1.01, 1.23) | 0.036 |
| Moderate/high distress (K10) | 1.16 (1.06, 1.27) | 0.001 |
| Mental illness previously diagnosed | 1.27 (1.16, 1.39) | 0.000 |
| Ever injected drugs | 1.68 (1.51, 1.86) | 0.000 |
| Ever overdosed | 1.4 (1.27, 1.53) | 0.000 |
| Low social support (ESSI < 28) | 1.17 (1.07, 1.28) | 0.000 |

aHR: adjusted hazard ratio; ASSIST: Alcohol Smoking and Substance Involvement Screening Test; K10: 10-item Kessler Psychological Distress Scale; ESSI: Enrichd Social Support Inventory

**References**

1. Krawczyk N., Eisenberg M., Schneider K. E., Richards T. M., Lyons B. C., Jackson K. et al. Predictors of overdose death among high-risk emergency department patients with substance-related encounters: a data linkage cohort study, Annals of emergency medicine 2020: 75: 1-12.

2. Colledge S., Peacock A., Leung J., Larney S., Grebely J., Hickman M. et al. The prevalence of non-fatal overdose among people who inject drugs: a multi-stage systematic review and meta-analysis, Int J Drug Policy 2019: 73: 172-184.

3. Degenhardt L., Peacock A., Colledge S., Leung J., Grebely J., Vickerman P. et al. Global prevalence of injecting drug use and sociodemographic characteristics and prevalence of HIV, HBV, and HCV in people who inject drugs: a multistage systematic review, The Lancet Global Health 2017: 5: e1192-e1207.

4. Butler T., Levy M., Dolan K., Kaldor J. Drug use and its correlates in an Australian prisoner population, Addiction Research & Theory 2003: 11: 89-101.

5. Milloy M. J., Wood E., Reading C., Kane D., Montaner J., Kerr T. Elevated overdose mortality rates among First Nations individuals in a Canadian setting: a population‐based analysis, Addiction 2010: 105: 1962-1970.

6. Australian Institute of Health and Welfare. The health and welfare of Australia's Aboriginal and Torres Strait Islander peoples 2015, Canberra: AIHW; 2015.

7. Fazel S., Bains P., Doll H. Substance abuse and dependence in prisoners: a systematic review, Addiction 2006: 101: 181-191.

8. Winter R. J., Young J. T., Stoove M., Agius P. A., Hellard M. E., Kinner S. A. Resumption of injecting drug use following release from prison in Australia, Drug and Alcohol Dependence 2016: 168: 104-111.

9. Keen C., Young J. T., Borschmann R., Kinner S. A. Non-fatal drug overdose after release from prison: a prospective data linkage study, Drug and Alcohol Dependence 2020: 206: 107707.

10. Winter R. J., Stoové M., Degenhardt L., Hellard M. E., Spelman T., Jenkinson R. et al. Incidence and predictors of non-fatal drug overdose after release from prison among people who inject drugs in Queensland, Australia, Drug and Alcohol Dependence 2015: 153: 43-49.

11. Mitchell P. H., Powell L., Blumenthal J., Norten J., Ironson G., Pitula C. R. et al. A short social support measure for patients recovering from myocardial infarction: the ENRICHD Social Support Inventory, J Cardiopulm Rehabil Prev 2003: 23: 398-403.

12. Kessler R. C., Andrews G., Colpe L. J., Hiripi E., Mroczek D. K., Normand S. L. et al. Short screening scales to monitor population prevalences and trends in non-specific psychological distress, Psychol Med 2002: 32: 959-976.

13. Andrews G., Slade T. Interpreting scores on the Kessler psychological distress scale (K10), Aust N Z J Public Health 2001: 25: 494-497.

14. Cumming C., Kinner S. A., McKetin R., Young J. T., Li I., Preen D. B. The predictive validity of the Alcohol Smoking and Substance Involvement Screening Test (ASSIST) for moderate‐to‐high risk cannabis, methamphetamine and opioid use after release from prison, Addiction 2023: 118: 1107-1115.
